# Evaluation of cardiopulmonary resuscitation quality during the pandemic of COVID-19

**DOI:** 10.1186/s12873-022-00754-x

**Published:** 2022-12-05

**Authors:** Yang Yu, Xiaojie Liu, Lijuan Wang, Yuchen Gao, Yao Ding, Hushan Ao

**Affiliations:** 1 Department of Anesthesiology, Fuwai Hospital, Chinese Academy of Medical Sciences & Peking Union Medical College, Beijing, China; 2 Department of Anesthesiology, The Affiliated Hospital of Qingdao University, Qingdao, China

**Keywords:** Cardiopulmonary resuscitation, Simulator, Evaluation, Online

## Abstract

**Background:**

Cardiopulmonary resuscitation (CPR) is an important technique of first aid. It is necessary to be popularized. Large-scale offline training has been affected after the outbreak of Coronavirus disease 2019 (COVID-19). Online training will be the future trend, but the quality of online assessment is unclear. This study aims to compare online and offline evaluations of CPR quality using digital simulator and specialist scoring methods.

**Methods:**

Forty-eight out of 108 contestants who participated in the second Chinese National CPR Skill Competition held in 2020 were included in this study. The competition comprised two stages. In the preliminary online competition, the contestants practiced on the digital simulator while the specialist teams scored live videos. The final competition was held offline, and consisted of live simulator scoring and specialist scoring. The grades of the simulator and specialists in different stages were compared.

**Results:**

There was no statistical significance for simulator grades between online and offline competition(37.7 ± 2.0 vs. 36.4 ± 3.4, *p* = 0.169). For specialists’ grades, the video scores were lower than live scores (55.0 ± 1.4 vs. 57.2 ± 1.7, *p* < 0.001).

**Conclusion:**

Simulator scoring provided better reliability than specialist scoring in the online evaluation of CPR quality. However, the simulator could only collect quantified data. Specialist scoring is necessary in conjunction with online tests to provide a comprehensive evaluation. A complete and standardized CPR quality evaluation system can be established by combining simulator and specialist contributions.

**Supplementary Information:**

The online version contains supplementary material available at 10.1186/s12873-022-00754-x.

## Introduction

Cardiac arrest (CA) is one of the major causes of death and an increasing health concern worldwide [1]. In one year, 356,500 Americans and 115,600 Japanese suffered out-of-hospital CA [2, 3]. The annual number of sudden deaths from CA in China was estimated from a regional survey at approximately 540,000 [4]. Cardiopulmonary resuscitation (CPR) is widely recognized as a central component of first-aid for CA [5]. According to the latest international guidelines, high-quality CPR with rapid defibrillation is a focal point in resuscitating adult and pediatric victims of CA [6]. The quality of CPR directly affects the survival rate and neurological outcome of CA resuscitation [7, 8]. If effective CPR is not started within four minutes after CA, the cerebral ischemic and hypoxic damage will be irreversible [9]. Although CPR was been included in basic first-aid training for decades, effective CPR administered by bystanders remains infrequent [10]. Universal CPR training still plays an essential role in the promotion of public health [11].

Coronavirus disease 2019 (COVID-19) is a collection of syndromes caused by SARS-CoV-2. Since December 2019, COVID-19 cases have been identified in all countries [12]. The World Health Organization has declared it a pandemic disease [12, 13]. The pandemic and the consequent lockdowns introduced major changes in behavior regarding work, cooperation, and learning [14]. Regular training is important in increasing willingness and confidence when performing CPR [15, 16]. The offline CPR courses were paused because of lock-down strategies. Among medical professionals, the CPR quality is known to decrease within only three months after training [5, 17]. Traditional CPR training combines face-to-face courses with practice on simulators. Trainers make real-time evaluations of quality and correct the mistakes of trainees. Online training has been evaluated as a potential substitute. Online remote qualifications with Internet and smart CPR simulators have been available for years [18, 19]. A randomized trial confirmed the practicality of offline objective CPR skill assessment by a simulator [20]. There remain limitations such as high cost, lack of supervision, fraud, and cheating in evaluations [21].

To evaluate the quality of online CPR training, a smart simulator and video scoring by remote specialists are both technically accessible. So far, few studies have described the effectiveness of online evaluation using the two techniques. In this study, we analyzed data obtained from National CPR Skill Competition held in China, to compare online (via video) and offline (face-to-face) evaluation of CPR quality using digital simulators and specialist scoring methods.

## Methods

### Participants and data

**Fig. 1:**
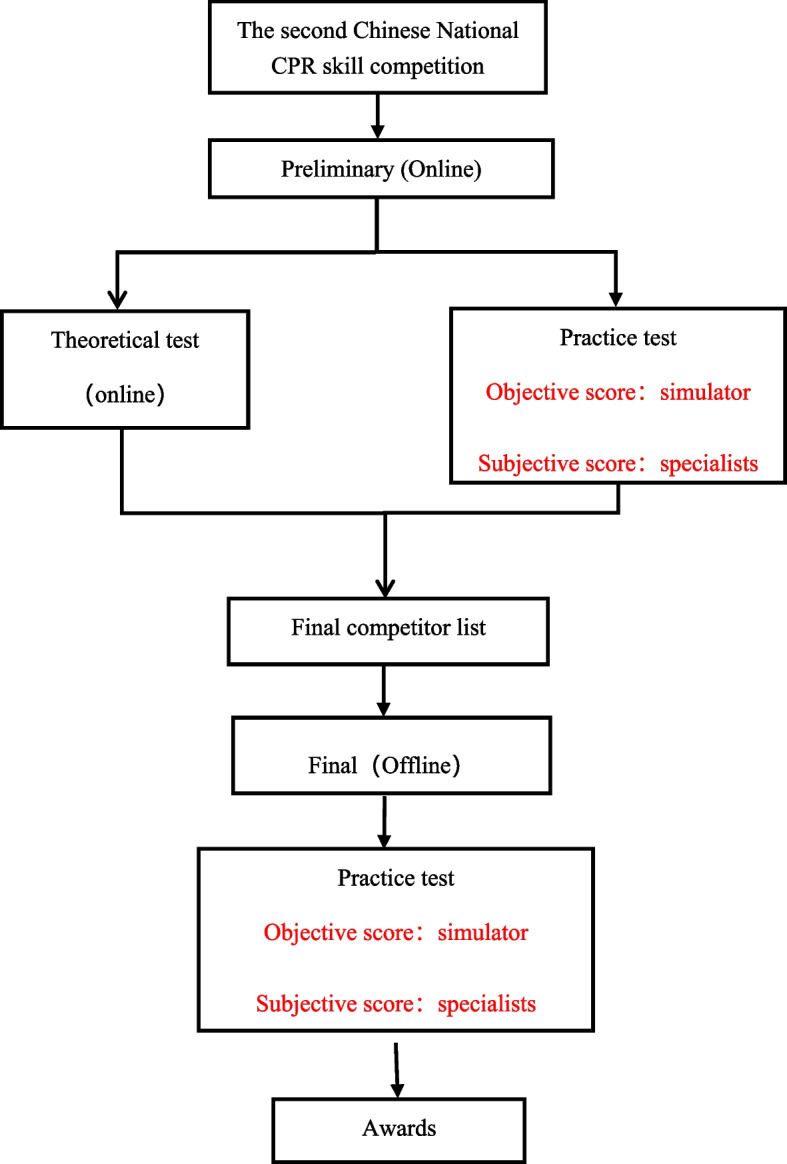
Competition flow chart

All contestants in the Second Chinese National CPR Skill Competition were included in this observational study. The Competition was hosted by the Chinese Society of Cardiothoracic and Vascular Anesthesiology (CSCVA). The contestants were medical professionals who received regular CPR training. They participated online in the preliminary competition in 54 separate standardized exam rooms around China on October 24th, 2020. Each contestant performed 5 rounds of standard CPR and one automated external defibrillator (AED) defibrillation procedure on a Laerdal Intelligent CPR simulator (174–01260, Resuscitation Anne QCPR, Laerdal, Norway). The sensors and scoring system attached to the simulator recorded real-time data and calculated the total score automatically. The scoring system criteria are presented in Table S1 of the Supplementary material. Simultaneously, a group of five CSCVA-certified CPR specialists monitored contestants with two Internet cameras and scored their performance on the criteria in Table S2 of the Supplementary material. The offline final competition (face-to-face competition) was held in Guangzhou on November 13th, 2020. Finals participants received no retraining after the preliminary competition. In an offline exam room, they performed the same procedures on the same model of the simulator, and the same specialist group observed and scored their performance on site. The competition flow can be seen in Fig. 1.

### Statistical analysis

Data are presented as mean ± standard deviation. Counts are by frequency and composition ratio. Repeated measures, paired sample T-test, analysis of variance, and non-parametric test were used to compare the differences in scores of the same players in the preliminary and the finals. *P* < 0.05 was considered statistically significant. All statistical analyses were performed using SPSS 25.0 software.

## Results

**Fig. 2:**
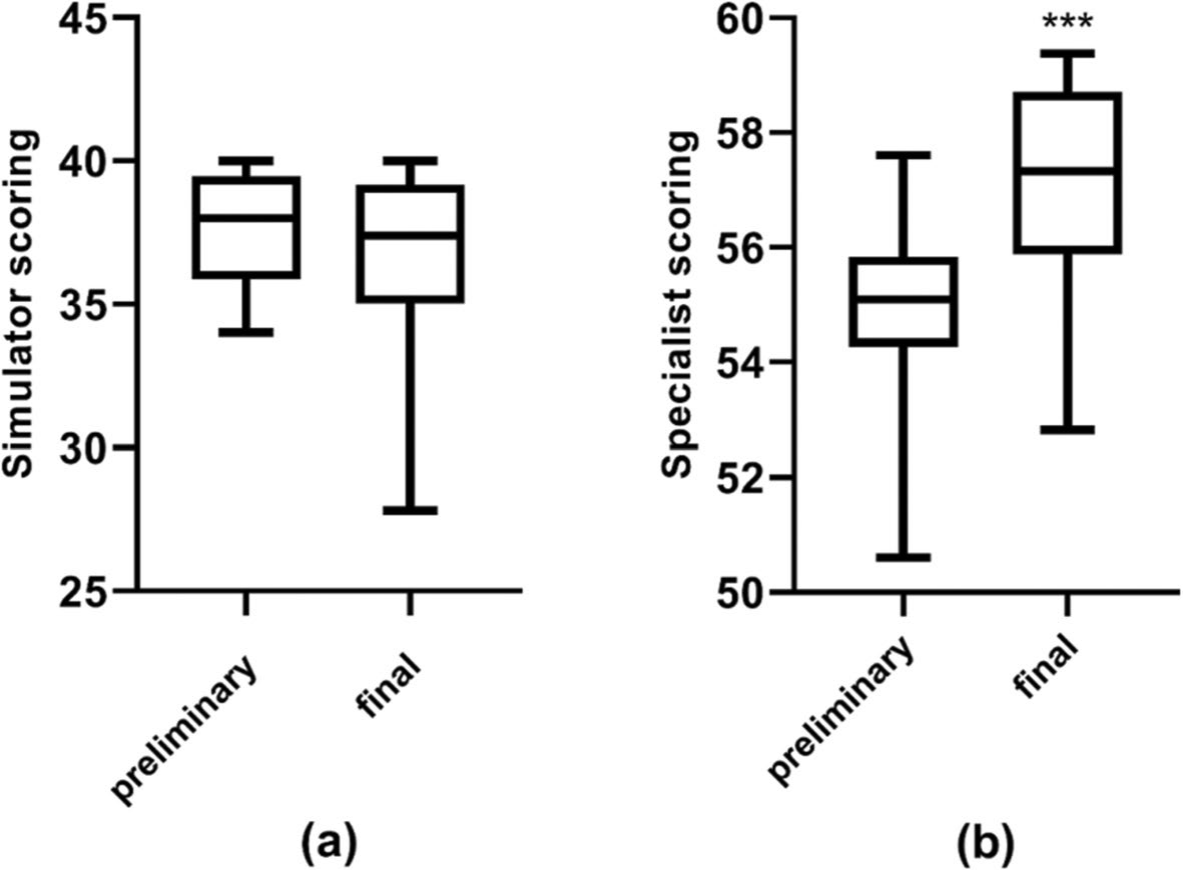
Box plots of participants’ scores. **a **Simulator scoring of practice in preliminary and final content. **b **Specialist scoring of practice in preliminary and final content

There were 108 contestants in the preliminary competition and 48 of them entered the final round. Among the 48 analyzed participants, 19 (40%) were men and 29 (60%) were women. The average age was 30 ± 4.9 years old, ranging from 22 to 44 years. For simulator scoring, the standard derivation of the online competition was 2.0 and offline was 3.4. The Kruskal-Wallis test showed no statistical significance between online and offline scoring (*p* = 0.169) (Table 1). For specialist scoring, the standard derivation of the online competition was 1.4 and offline was 1.7. The online and offline scores were statistically different with a paired sample t-test (*p* < 0.001) (Table 1). Such variation in data stability could also be seen in Fig. 2.

**Table 1:** The difference in scoring between evaluation methods

Evaluation method	Preliminary (online)	Final (offline)	*P*-value
Simulator scoring	37.7 ± 2.0	36.4 ± 3.4	0.169
Specialist scoring	55.0 ± 1.4	57.2 ± 1.7	< 0.001

## Discussion

The present study analyzed the scores of the contestants in a hybrid CPR skill competition comprising online and offline components. We found no statistical difference between online and offline evaluations of CPR quality based on sensors and programs on a digital simulator. The specialist scoring is significantly different between online and offline evaluations. The offline contestants tended to get higher scores from the specialist scoring system. However, we found that the simulator scores had higher standard derivation, this may be related to the state of competitors on the day and simulators will give higher resolution scoring. Also, this will reveal that simulator scoring may be more objective than specialist scoring.

The most critical maneuvers affecting a patient’s prognosis in CPR are compression depth and frequency, both of which are quantifiable indicators [5]. An instrumented direct feedback device measures compression rate, depth, hand position, recoil, and chest compression fraction and provides real-time audio or visual feedback (or both) on these critical CPR skills [22]. Training for chest compressions based on the use of real-time feedback software (Laerdal QCPR) guided by an instructor is superior to instructor-guided feedback training in terms of overall chest compression technical skill acquisition [11]. Therefore, the CPR quality evaluation system is effective for simulator scoring. However, the simulator is too sensitive: a slight change in the subject’s action may lead to fluctuations in the results. So it is not enough solely rely on machines.

In our study, practice quality evaluation in both online and offline modes did not affect the objective results obtained by the simulator evaluation. Therefore, it is essential for the evaluation of the quality of CPR on an objective basis. The more common specialist scoring approach also is more subjective. Even if the average score of five specialists is adopted, differences remain. Therefore, the subjective scores obtained by the judges should be considered a supplement to the objective scores, such as compression depth and frequency.

Medical simulations have great promise to provide training at less cost and without risk to patients. However, these advantages are not sufficient to conclude effectiveness [23]. A study showed that simulator assessment can be sufficient but they also suggest a combination of assessment tools will be much better [24]. This study analyzing two competitions with the same contestants found the offline specialist scoring to be significantly higher than the online specialist scoring. Explanations could reflect on both players and the specialists: Players sensed the offline finals as more important than online, making the onsite competition more urgent, preparation more extensive, and the competition mode more familiar. The onsite performances were more stable than in the preliminary round, and the results are slightly higher. Specialists applied the same scoring standards for the preliminary and final rounds. And the judges were all senior professors. Although there are three screens for online viewing, the details may not have been clear enough. Plus, the atmosphere of the finals and the neatness of the players’ clothing could cause specialists to subjectively increase emotional points.

This is consistent with the conclusion of the article published by Camilla Hansen and others in 2019. They believe that the basic life support (BLS) certified instructors still have a poor assessment of the quality of CPR, and the compression depth and artificial respiration are not clear [25]. Machines can ensure quality through a quantifiable index. Specialists can objectively integrate many factors to ensure the stability of the score. We need to combine these and establish a new quality evaluation system for online training.

The results of this article suggest that the current CPR quality evaluation system should not depend only on specialist scoring, and that differences in competition scenes will significantly affect the specialist scoring. Human judgments are susceptible to error due to a host of factors, from fatigue to various biases of judges [26]. Specialist scoring can be used as a supplement to the simulator scoring, and indicators that cannot be quantified and monitored, such as whether the practice posture is standardized, whether the arm is completely perpendicular to the ground when pressing, etc. Due to the high price of current simulators with evaluation functions, it is difficult to train enough people on CPR. Thus, adopting new approaches, technology, and further research are necessary.

CPR is the most effective rescue method for patients with CA. The quality of CPR is low during both in-hospital and out-of-hospital rescue [27]. The domestic penetration rate of CPR training is lower in China than in other countries [28]. As populations are, the prevalence of cardiovascular diseases is increasing. To achieve a rapid increase in the penetration rate in the short term and improve the quality of CPR, online training with feedback devices is the most apparent path.

The American Heart Association(AHA) statement on resuscitation education science emphasizes the importance of using objective data to improve BLS and advanced life support skills. The 2015 European CPR Guidelines also recommend the use of feedback devices to improve the quality of CPR [11]. However, feedback devices may be prohibitively expensive and not available in all situations. There is currently no other objective method to assess the quality of CPR [25].

Studies have shown that training with assistive devices can improve the quality of CPR [20]. Online training will be an inevitable trend in the post-epidemic era. CPR requires regular retaining to ensure the quality of operation. Online training can facilitate this. The AHA guidelines also suggest that continuous enhancement of this skill in the short term can improve the quality of practice [17]. Even doctors who have received multiple pieces of training should retrain regularly to improve the quality of their practice and ensure clinical safety.

This study has several limitations. Because of the influence of COVID-19, it is not possible to organize large-scale activities for comparison the sample size was lacking. All the participants come from different medical institutions and their educational backgrounds differed, which may have influenced comparisons. As this study only involved medical staff, the results apply only to that occupation.

## Conclusion

Online CPR quality evaluated by digital simulators can better reflect the actual performance level of trainees, and be consistent with the traditional offline evaluation. Simulators can be used as an important means of monitoring training in online CPR training. Standardized CPR quality evaluation should advance beyond the traditional evaluation model with a quality evaluation system established on objective results obtained after the quantitative evaluation of the cardiopulmonary resuscitation simulator and supplemented by the subjective results of evaluators.

## Supplementary Information

**Additional file 1: Table S1.** The criteria of scoring system in simulator. **Table S2.** The criteria of specialist scoring.

## Data Availability

The datasets used and/or analyzed during the current study are available from the corresponding author upon reasonable request.

## Abbreviations

CPR: Cardiopulmonary Resuscitation
COVID-19: Coronavirus disease 2019
CA: Cardiac arrest
CSCVA: Chinese Society of Cardiothoracic and Vascular Anesthesiology
AED: Automated external defibrillator
BLS: Basic life support
AHA: American Heart Association
