# Supplementary material for: Evaluation of cardiopulmonary resuscitation quality during the pandemic of COVID-19
Source: BMC Emerg Med. 2022 Dec 5;22:193. doi: 10.1186/s12873-022-00754-x (PMC9724342; doi:10.1186/s12873-022-00754-x)
Supplement: Supplementary file 1 — Additional file 1: Table S1. The criteria of scoring system in simulator. Table S2. The criteria of specialist scoring. [file 12873_2022_754_MOESM1_ESM.docx]

**Table S1.** The criteria of scoring system in simulator.

| No. | Item | Requirement | Score |
| --- | --- | --- | --- |
| 1 | Starting time of CPR | The first chest compression should start in 30 seconds from the beginning of procedure | 2 |
| 2 | Preparation time of AED | The AED should be opened and prepared in 20 seconds | 2 |
| 3 | Finish time of electric defibrillation | This part should be finished in 20 seconds | 2 |
| 4 | Sampling interruptions Duration of chest compressions | Less than 10 seconds | 2 |
| 5 | Time taken for 5 cycles of CPR after defibrillation | Time limit between 110~120 seconds to complete | 2 |
| 6 | Average compression depth | 5-6cm | 20 |
| 7 | Correct compression frequency | 100~120 times/min |  |
| 8 | Total cumulative compressions | 150 times |  |
| 9 | Insufficient press force error times | ___ |  |
| 10 | Number of errors found at the pressing site | ___ |  |
| 11 | Thorax not fully relaxed error times | ___ |  |
| 12 | Total cumulative ventilation | 10 times | 20 |
| 13 | Number of hyperventilation errors | ___ |  |
| 14 | Number of under-ventilation errors | ___ |  |
| 15 | Number of errors in ventilation too fast | ___ |  |
| 16 | Ratio of compressional ventilation | 30:2 | 3 |
| 17 | Completed 5 rounds | ___ |  |

**Table S2. The criteria of specialist scoring**

| No. | Item | Requirement | Score |
| --- | --- | --- | --- |
| 1 | Preparation | The contestant should wear gloves and make self-protection and start procedure after the sign | 1 |
| 2 | Environment evaluation | The contestant should confirm the safety of environment | 2 |
| 3 | Judgmental Awareness | Head down call / 1 time each on the left and right / painful stimulation such as shoulder tapping | 2 |
| 4 | Shouting for help | Incoming resuscitation/activate EMS/carry manual defibrillator | 1 |
| 5 | Supine position | Untie the top, pad the backboard/smooth out the body (just dictate) |  |
| 6 | Determination of respiration, heartbeat | Touch the carotid artery / side view of the chest / tour of the limbs / count time 7 seconds | 1 |
| 7 | Compression (10 times) | Correct posture, no impact compressions,Smooth rhythm | 5 |
| 8 | Manual electroshock defibrillation | Correct process | 12 |
| 9 | Open airway | Handwriting specifications, clearing the mouth, | 5 |
| 10 | Artificial ventilation | E-C Method | 5 |
| 11 | 5 rounds of CPR | Visual assessment of operational quality | 25 |
| 12 | Re-examination and evaluation | After five rounds of CPR, check for circulatory signs and spontaneous breathing and count for 10 seconds | 2 |
